# Binocular vs. monocular 3D cues in multiple object tracking: expertise differences between soccer players and non-athletes

**DOI:** 10.1186/s41235-025-00658-x

**Published:** 2025-07-26

**Authors:** Xiang Che, Jiayue Ma, Yu Zhang, Chen Zhou, Qian Zhou, Kun Zhang, Jijun Lan, Qi Hui, Jie Li

**Affiliations:** 1 College of Physical Education, Northwest Normal University, Lanzhou, China; 2 School of Psychology, Shaanxi Normal University, Xi’an, China; 3 Shaanxi Vocational & Technical College, Xi’an, China; 4 School of Psychology, Beijing Sport University, Beijing, China; 5 School of Physical Education, Shaanxi Normal University, Xi’an, China; 6 School of Management, Xi’an University of Science and Technology, Xi’an, China; 7 Center for Cognition and Brain Disorders, The Affiliated Hospital, Hangzhou Normal University, Room 409, No. 27, Shuyuan Building, Cangqian Campus, Hangzhou, 311121 China; 8 Institutes of Psychological Sciences, Hangzhou Normal University, Hangzhou, China

**Keywords:** Three-dimensional MOT, 3D depth cues, Depth perception, Soccer players

## Abstract

Classical two-dimensional multiple object tracking (2D-MOT) measures the cognitive ability to track multiple moving elements in real-life-like scenarios. Stereo-three-dimensional MOT (S-3D-MOT), a more ecologically valid form of 2D-MOT, shows better tracking performance in soccer players. Its unique feature is the additional binocular and monocular 3D cues compared to 2D-MOT, but their individual contributions to MOT performance are unclear. To fill this research gap, the current study introduced a three-dimensional MOT task on a flat screen (F-3D-MOT) to distinguish the roles of binocular and monocular 3D cues. F-3D-MOT provides additional monocular 3D cues compared to classical 2D-MOT but lacks binocular 3D cues compared to S-3D-MOT. Moreover, whether the effects of these 3D cues on MOT performance vary between soccer players and non-athletes remains unclear. Therefore, both groups were recruited for this study. The results showed that soccer players performed significantly better than non-athletes specifically in S-3D-MOT, indicating their enhanced sensitivity to binocular 3D cues. In contrast, neither monocular cues (F-3D-MOT) nor 2D displays led to significant differences between the two groups.

## Introduction

Multiple Object Tracking (MOT), initially introduced by Pylyshyn and Storm (1988), is a task that gauges an individual’s cognitive capacity to track multiple moving objects, like people and vehicles. Owing to its paradigm’s alignment with real-world activities such as driving and team sports, it has drawn the attention of numerous researchers (Meyerhoff et al., 2017).

The traditional MOT tasks are presented in a two-dimensional (2D) form and involve three stages: designating the objects to be tracked, tracking them, and choosing the initially designated objects. On a flat-screen personal computer, identical targets (usually limited to four) and distractors are randomly arranged (Pylyshyn & Storm, 1988; Scholl et al., 2001).

Nevertheless, there exists another MOT task, the 3D-MOT task. Here, stimuli are presented in a three-dimensional (3D) graphic structure and demand binocular vision for tracking, enabling a more accurate portrayal of 3D scenes in real-life scenarios (Faubert & Sidebottom, 2012; Parsons et al., 2014). Due to the marked differences in presentation and motion trajectories between 3D-MOT and 2D-MOT, direct comparison between them is arduous. Che et al. (2023) discovered, through an adaptive staircase procedure, that female soccer players exhibited a higher speed threshold in 3D-MOT than in 2D-MOT, uncovering for the first time the tracking disparities between these two MOT task modes within the soccer domain.

In 3D-MOT, compared to 2D-MOT, the 3D environment offers supplementary 3D cues. Prior 3D-MOT studies were carried out in a stereo environment where stimuli were constructed using both monocular 3D cues (e.g., 3D graphic structures) and binocular 3D cues (e.g., in-depth motion processing), which are vital visual functions for functioning in the 3D world (Cormack et al., 2017). Monocular 3D cues in 3D stimuli signify that only the 3D graphic structure presents 2D perspective projections of 3D environments (Tavanti & Lind, 2001), originating from alterations in 3D coordinate system positions, texture, and luminance gradients (Bingham & Pagano, 1998). Binocular 3D cues, conversely, emerge when an object moves in a real 3D environment, as the two eyes process slightly different information in the visual system, offering cues to depth and motion in depth (Harris et al., 2008), and can assist in perception and in determining the speed of motion in depth (Harris & Watamaniuk, 1995).

While 3D-MOT encompasses both binocular and monocular 3D cues, 2D-MOT lacks 3D cues. One potential reason for female soccer players’ superior performance in 3D-MOT compared to 2D-MOT could be their utilization of these 3D cues (Che et al., 2023). Vidakovic and Zdravkovic (2010) explored the role of monocular 3D cues such as relative size, contrast, and texture gradient and found that, on a flat PC screen within a simulated 3D environment, only contrast 3D cues (gray at the top and black at the bottom) contributed to MOT tracking performance, rather than other 3D cues or all three combined. However, the distinct roles of binocular and monocular 3D cues in 3D-MOT on tracking performance remain ambiguous. In other words, further research is needed to determine whether the better tracking performance in 3D-MOT compared to 2D-MOT is more attributable to monocular 3D cues or binocular 3D cues. Manipulating different categories of 3D cues between 2D-and 3D-MOT tasks may help resolve the cue-effect issues in Che et al. (2023) and clarify the tracking mechanism in 3D-MOT.

In team sports like basketball, the 3D-MOT tracking speed threshold of National Basketball Association (NBA) players is positively correlated with assist and steal indicators and lower turnovers in the subsequent season (Mangine et al., 2014). 3D-MOT has been regarded as an effective perceptual-cognitive training tool in the team sports domain (Mangine et al., 2014; Romeas et al., 2016; Scharfen & Memmert, 2021) because its cognitive processing mirrors that of a real soccer game (Faubert & Sidebottom, 2012; Parsons et al., 2014). Perceptual-cognitive skills, which are crucial for athletes’ game performance (Voss et al., 2010), are more accurately measured with more realistic paradigms (Mann et al., 2007). However, some studies have indicated that the training effect of 3D-MOT is not as effective as anticipated (Harris et al., 2020), even in the soccer domain (Harenberg et al., 2021). Vater et al. (2021) noted that the evidence for the far-transfer training effect of 3D-MOT is too feeble to justify replacing sport-specific training time. Recently, Romeas et al. (2025) revealed that while dual-task 3D-MOT training enhanced task-specific speed thresholds by 56% in youth soccer athletes, these improvements failed to transfer to either attentional performance or on-field success metrics. Thus, further exploration of the mechanism between classic 2D-MOT and 3D-MOT is necessary to understand how the training effect is generated.

Individuals vary in cognitive abilities. Non-athletes may not always need the MOT ability that is essential for team-sport players. These differences may be linked to genetic talent or training-derived expertise. A meta-analysis study has compared MOT tracking performance between experts and non-experts in tracking multiple elements (Liu et al., 2024). For example, radar operators, who must follow multiple signals on a monitoring system, display higher accuracy in tracking multiple objects (Allen et al., 2004). However, since radar monitor systems are presented on a flat PC screen, 2D-MOT suffices to measure the cognitive-skill advantages of the expert group. In team-sports domains, athletes need to identify and track the positions of teammates and opponents in a 3D environment using depth perception. Therefore, comparing the 3D-MOT ability between soccer players and non-athletes is more effective than other MOT tasks in revealing the tracking advantages of soccer players and can offer a deeper understanding of the 3D-MOT mechanism in different individuals.

Overall, while previous research has made some progress in understanding the role of stereo cues in MOT, as demonstrated by Plourde et al. (2017) in age-related studies and Zhang et al. (2025) in young athletes, the distinct roles of binocular and monocular 3D cues have still not been comprehensively explored. As a result, how these cues respectively affect the MOT performance remains unclear. To address these issues, this study isolated binocular and monocular cues by adding a simulated three-dimensional MOT task on a flat screen, building on the work of Che et al. (2023). This study included an MOT task with only monocular 3D cues (flat-3D-MOT, F-3D-MOT), an MOT task with both monocular and binocular 3D depth cues (stereo-3D-MOT, S-3D-MOT), and an 2D-MOT task with no 3D cues.

To further explore the 3D-MOT tracking mechanism in the soccer domain, this study recruited both ordinary college students as a non-athlete group and soccer players from a university team as an expert group. Comparing these two groups may clarify the contribution of each depth cue in different groups. Given that soccer players can utilize 3D cues of teammates and opponents on the soccer field during games, this study hypothesizes that soccer players will outperform non-athletes in both F-3D-MOT (monocular) and S-3D-MOT (binocular).

## Materials and methods

### Participants

A power analysis (G*Power; effect size, *f* = 0.25) indicated that a total sample of 56 participants would yield at least 0.80 power at α = .05. The sample consisted of 28 (male = 10, female = 18) university students as the non-athlete group aged 18 to 26 years (*M* ± *SD* = 22.68 ± 2.20 years), and 29 (male = 12, female = 17) university soccer players as the soccer players’ group aged 18 to 23 years (*M* ± *SD* = 20.65 ± 1.22 years). Participants in the non-athlete group reported that they had no regular exercise activities. The participants in the soccer players’ group experienced years of soccer training (*M* ± *SD* = 8.37 ± 2.40 years), attended weekly training sessions (*M* ± *SD* = 8.79 ± 1.08 h), and qualified for national level I or II according to the General Administration of Sport of China. According to the athletes’ expertise classification system of Swann et al. (2015), the eliteness of soccer players’ group can be classified at a semi-elite level. All the participants (including both groups of soccer players and non-athletes) have no laboratory MOT task experience. Course credit was provided as a participation reward. All the participants had normal vision or corrected to normal vision and signed a written informed consent form. The study was conducted following the Declaration of Helsinki, and the protocol was approved by the Ethics Committee of Shaanxi Normal University (No. 202316001).

### Apparatus and stimuli

#### S-3D-MOT

All MOT tasks were performed using the Unity™ engine. The S-3D-MOT task was administered via an HTC Vive Pro2 head-mounted display (HMD) within a SteamVR environment, providing a 110° field of view, combined resolution of 4896 × 2448, and 120 Hz refresh rate. The pupil spacing could be adjusted for each subject to ensure all participants can see the S-3D-MOT task. Participants were instructed to use the HTC Vive Controller to complete the S-3D-MOT task in a VR environment.

**Fig. 1:**
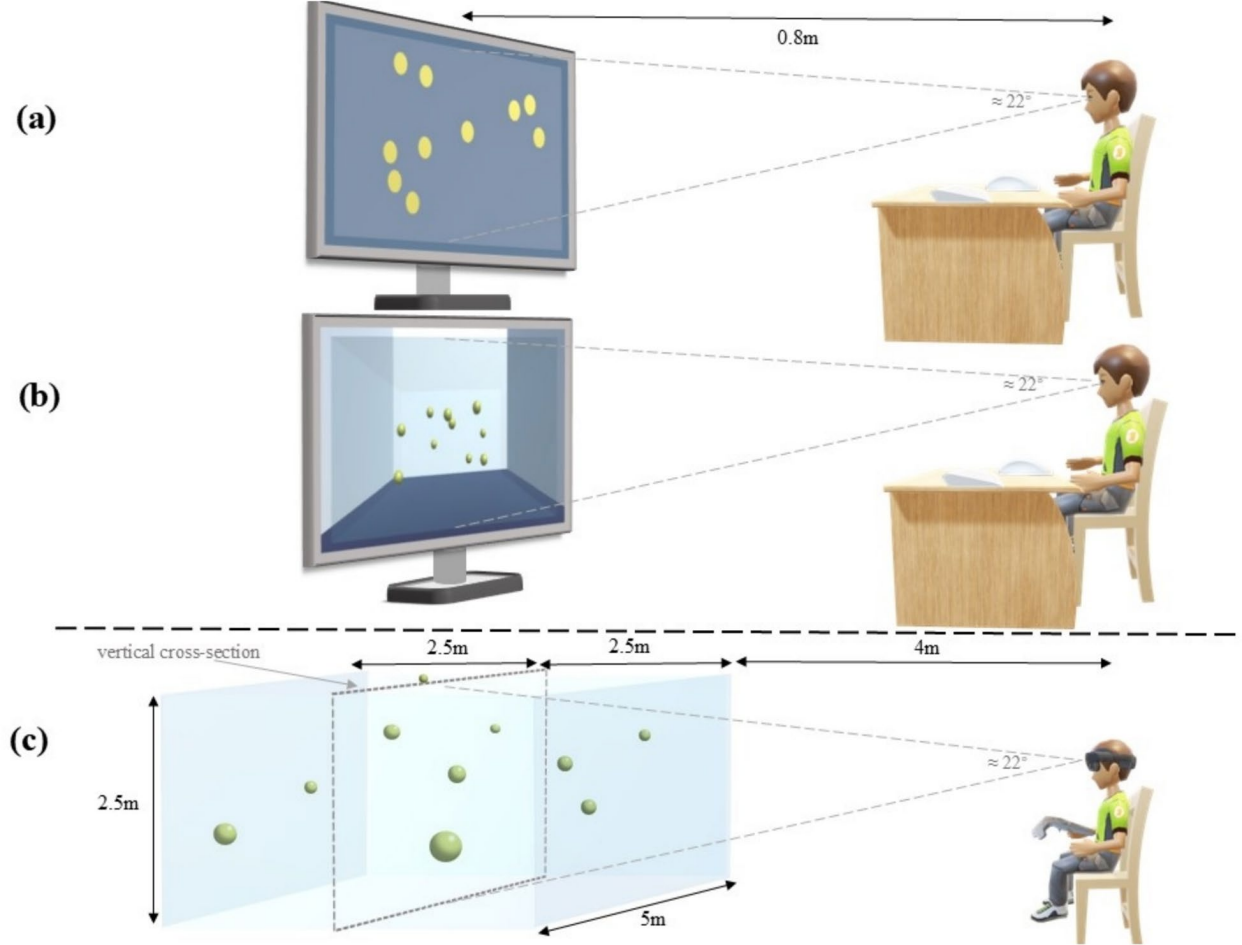
The charts above indicate the task presentation of **a** 2D-MOT, **b** F-3D-MOT, and **c** S-3D-MOT. The 2D-MOT task includes no 3D cues. The F-3D-MOT includes only monocular cues in depth. Moreover, the S-3D-MOT includes both binocular and monocular cues in depth

S-3D-MOT task adopted in the HMD VR environment, similar to Che et al. (2023), we designed a virtual rectangular-shaped room with a depth of 5 m, a width of 5 m, and a height of 2.5 m (Fig. 1). The room had white walls and a blue floor. The HMD was located 4 m away from the nearest boundary of the room. The stimulus objects (3D yellow spheres) moved randomly in the virtual room space. The vertical cross-Sect. (5 m wide and 2.5 m in height, 6.5 m to participants) was defined along the midline of the length of the room, dividing the room evenly into two equal spaces. Therefore, regardless of how the stimulus objects move in the virtual 3D room, their average visual angle projected onto this vertical interface is consistent. The vertical cross-section in S-3D-MOT has a horizontal (≈ 42°) and vertical visual angle (≈ 22°).

Each object in the S-3D-MOT in the VR environment had a diameter of 27.94 cm (≈ 2.5° average) and an identical initial motion speed of 0.68 m/s (≈ 6°/s). In S-3D-MOT, a baby blue filter was added to all six sides of the virtual room’s cuboid. Additionally, the binocular depth information was thus controlled. To make objects percept as 3D spheres in the S-3D-MOT task, we provide monocular 3D information, a light source, face to the objects at the upper right location. The Objects motion trajectory in S-3D-MOT is such that if a collision occurred while tracking, all the moving objects bounce off the walls or bounce off each other.

In the S-3D-MOT condition, we calculated binocular disparity underpinning the binocular 3D cues’ influence on MOT task performance. Calibrated by object-background depth, observer’s viewing distance, and interpupillary distance (IPD), the binocular disparity range was set at a minimum of 0° for the closest objects and, a maximum of 0.22° for the farthest. These values, crucial for depth perception, were calculated by formula (Epstein, 1977; Howard & Rogers, 2012):

$$\Delta = \frac{d \times b}{{D \times D}}$$

$\Delta$ is the binocular disparity, *d* = 2.5m (the distance from the vertical cross-section to the farthest and nearest points of the object), *b* is the IPD, and *D* = 6.5m (the distance from the observer to the vertical cross-section). We brought the average IPD as 6.5cm or 0.065m around 18–23-year Chinese participants in the formula (Shi et al., 2011).

#### F-3D-MOT

The 2D-MOT and F-3D-MOT tasks on the PC device were presented on a 27-inch monitor with a resolution of 1920 × 1080. Participants were instructed to use a mouse and keyboard to select and confirm the correct object to complete both 2D-MOT and F-3D-MOT tasks on the PC.

The stimuli present in F-3D-MOT are calculated by all the stimuli projected to the vertical cross-section of the virtual room from the S-3D-MOT task. F-3D-MOT and 2D-MOT tasks were presented with an identical horizontal and vertical visual angle with the vertical cross-section in S-3D-MOT (horizontal ≈ 42°, vertical ≈ 22°). These visual angles of objects’ size and initial motion speed (diameter of 27.94 cm ≈ 2.5° average, initial motion speed of 0.68 m/s ≈ 6°/s) had also been set in F-3D-MOT and 2D-MOT tasks, same as in S-3D-MOT.

For enhanced comparability between the 2D-MOT task and 3D conditions, in F-3D-MOT, a rectangular area was defined and a white filter was incorporated within the dark blue background, restricting the stimulus objects’ motion. In F-3D-MOT, a baby blue filter was added to all six sides of the virtual room’s cuboid, and to make objects percept as 3D spheres, we provide monocular 3D information, a light source, face to the objects at the upper right location, similar to S-3D-MOT. Objects motion trajectory is similar to both S-3D-MOT and F-3D-MOT. If the collision occurred while tracking, all the moving objects bounce off the walls or bounce each other. All objects in the F-3D-MOT were presented on a computer screen in a simulated 3D manner, similar to S-3D-MOT in VR. The F-3D-MOT task includes only monocular 3D cues.

#### 2D-MOT

The 2D-MOT task presented on a blue background. There were 10 randomly arrayed objects (flat yellow discs) in each trial, and four objects were designated as targets by flashing for 2-s. Second, participants were required to track all targets while all objects randomly moved for a fixed 8-s period. Third, participants selected targets and were presented with corrected feedback. Feedback for the four correct objects was indicated by a purple-to-yellow color change until the beginning of the next trial.

A rectangular area was defined within the blue background, and a white filter was added to 2D-MOT for enhanced comparability with the 3D conditions. The 2D-MOT task includes no 3D cues. Stimuli in the 2D-MOT task require the use of a mouse to select targets and press the SPACEBAR on the keyboard for submission.

In the 2D-MOT condition, objects moved on a 2D plane without occlusion or crossover, with collisions limited to interactions between objects and virtual walls. In contrast, both S-3D-MOT and F-3D-MOT allowed depth-dependent object interactions—collisions occurring when objects were in the same depth plane and crossovers when in different depths—along with collisions with virtual walls. Notably, S-3D-MOT uniquely enabled stereoscopic disambiguation of crossovers through binocular depth cues.

By systematically manipulating the presence and combination of monocular and binocular 3D cues across the 2D-MOT, F-3D-MOT, and S-3D-MOT tasks, we could isolate and clarify their distinct effects on multiple object tracking (MOT) performance. Specifically, the 2D-MOT task, devoid of any 3D cues, served as a baseline, while the F-3D-MOT task with only monocular 3D cues and the S-3D-MOT task with both monocular and binocular 3D cues allowed for a direct comparison (Table 1). This experimental design enabled us to determine how each type of 3D cue influenced participants’ ability to track multiple objects, providing insights into the underlying mechanisms of visual attention and spatial perception in MOT tasks.

**Table 1:** Monocular and binocular 3D cues in all three tasks

Modes	Monocular 3D cues		Binocular 3D cues	
	Designating targets-2 s	Tracking-8 s	Designating targets-2 s	Tracking-8 s
2D-MOT	–	–	–	–
F-3D-MOT	3D graphic structure, perspectivity, texture gradient	Objects overlapping, motion parallax	–	–
S-3D-MOT	3D graphic structure, perspectivity, texture gradient	Objects overlapping, motion parallax	Binocular disparity	Inter-ocular velocity differences

3D graphic structure: Use of three-dimensional shapes to suggest volume and spatial position

Perspectivity: Objects further away appear smaller, with parallel lines converging to indicate distance

Texture gradient: Textures become less distinct and more densely packed as objects recede

Objects overlapping: Occlusion where one object blocks another, indicating relative closeness

Motion parallax: Closer objects appear to move faster across the visual field than distant ones

Binocular disparity: Difference in an object’s position as seen by each eye, crucial for stereoscopic depth

Inter-ocular velocity differences: Variations in object motion speed perceived by each eye, aiding depth perception

### Experimental design and procedure

The effect of 3D graphic structures and motion trajectory in depth were manipulated using the factors of different groups and modes. The present study adopted a 2 (groups: non-athlete, soccer player) × 3 (modes: 2D, F-3D, S-3D) within-between-factor design. All participants completed all three MOT tasks in the VR and PC environments in Latin square order.

**Fig. 2:**
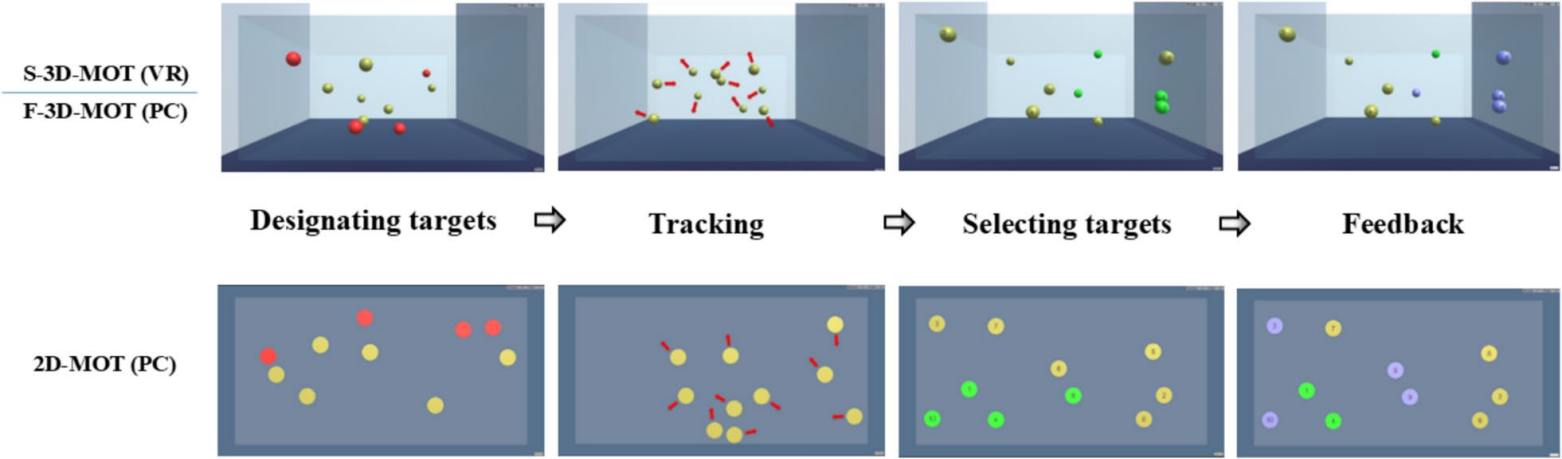
The chart indicated the procedure of each MOT task under modes (2D, F-3D, S-3D)

All participants adopted three MOT tasks with a staircase one-up one-down procedure and were instructed to complete 30 trials in each MOT task (Fig. 2). For the S-3D-MOT and F-3D-MOT, we introduced a practice session consisting of five trials to confirm that participants grasped the task requirements. Initially, each trial commenced with 10 objects (3D yellow spheres) positioned haphazardly within the virtual environment. Upon the participant activating the hand controller’s trigger, four out of the 10 objects started to flash red for a duration of 2 s, signifying the objects to be monitored. Subsequently, these four objects reverted to yellow, and all 10 objects began to move randomly within the virtual space for 8 s. During their movement, the objects would randomly alter their direction upon encountering the boundaries or colliding with one another. Participants were directed to monitor the four designated targets throughout the entire duration. At the end of the movement, all objects ceased their motion. Participants were then tasked with selecting and clicking on the four objects they had been tracking using the hand controller. The feedback for correctly identified objects was displayed.

The raw data were transferred from each trial to the speed ratio, indicating the change in the object’s motion speed compared to its initial speed (first trial). The speed ratio was calculated by the present object motion speed divided initial object motion speed (first trial, 0.68 m/s), in agreement with the adopted adaptive staircase procedure to investigate MOT performance (Faubert, 2013; Hu et al., 2022; Romeas et al., 2016). If participants correctly tracked all four balls, the motion speed increased by 30% over the speed in the previous trial. For instance, if the initial speed was 0.68 m/s and the participant succeeded in the first trial, the speed in the second trial would be 0.68 × 1.3 = 0.884 m/s. This increment challenges the participant further, aiming to push them closer to their performance limit. However, if participants made errors, the motion speed decreased by 20%. So, if the initial speed was 0.68 m/s and the participant failed the first trial, the speed in the second trial would be 0.68 × 0.8 = 0.544 m/s. This reduction helps the participant regain accuracy and find the appropriate level of difficulty. Suppose a participant has the following sequence of correct (C) and incorrect (I) last 10 of 30 trials: C, C, I, C, I, C, I, I, C, C. The speed ratio threshold was calculated from the average value of the last four inversion pairs of trials (i.e., transitions between correct and incorrect trials, or vice versa). Specifically, the last four pairs in this example are C-I, I-C, C-I, I-C (Faubert & Sidebottom, 2012). This threshold serves as an indicator of near-maximum MOT ability (Faubert & Sidebottom, 2012; Tullo et al., 2018).

Additionally, this study aims to replicate and extend the findings of Che et al. (2023). We adopted several experimental parameters consistent with their research, including the calculation of the speed ratio and the analysis of performance across trial bins. The rate of change in speed per trial (speed ratio) indicates different tracking speeds over 30 trials. This value represents the tracking speed information over time for both the MOT tasks on different devices and modes. The speed ratio, calculated as the present object motion speed divided by the initial speed (0.68 m/s), quantifies tracking speeds across 30 trials. For example, if a trial’s speed increased to 0.884 m/s (post-correct trial boost), the ratio would be 0.884 / 0.68 = 1.3, indicating 1.3-fold speed increase. This metric reflects performance-based speed adjustments and standardizes task difficulty across participants.

To further analyze the MOT tracking ability differences between two groups, we calculated the average tracking speed ratio for three distinct trial bins: the first (trials 1–10), the middle (trials 11–20), and the last bin (trials 21–30) by dividing the total of 30 trials into these segments. Trial bins provide dynamic evolution of tracking speed across all MOT tasks under different group and mode conditions, offering complementary insights to speed ratio thresholds. The average speed ratio is computed by dividing each trial’s speed in the bin by the initial speed (0.68 m/s), then averaging these individual ratios across all trials in the bin. The speed ratio changes across trials also provide valuable insights into the MOT ability differences between the two groups. In each MOT task, the trial bins were continuous with no breaks within a task, and a 2-min break was provided between each task. The total time for a participant to complete all tasks is about 27 min.

## Results

**Fig. 3:**
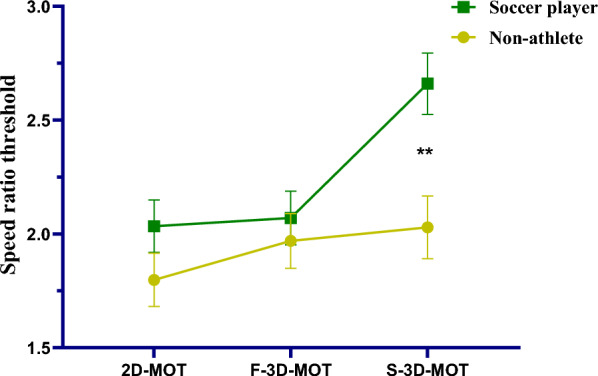
The chart above indicates the speed ratio threshold in groups (soccer players, non-athletes) and modes (2D, F-3D, S-3D). Error bars represent ± 1 SEM in all charts of the present study. **p < .01

We submitted the between-participants factor of Groups (non-athlete, soccer player) and within-participants factor of three Modes (2D, F-3D, S-3D) on the speed ratio threshold into a mixed-design repeated-measures analysis of variance (ANOVA) using SPSS (Statistical Package for Social Sciences, version 26.0, IBM, USA). There was a significant interaction between Groups and Modes (*F* (2, 110) = 5.097, *p* = .008, η_p_^2^ = 0.085). Post-hoc analysis indicated soccer players performed higher speed ratio threshold in S-3D-MOT task than non-athlete group (*M*_Soccer player_ = 2.660, *SE* = 0.135,* M*_Non-athlete_ = 2.029, *SE* = 0.138, *p* = .002; See Fig. 3), compared to there was no significant difference in F-3D-MOT task (*M*_soccer player_ = 2.070, *SE* = 0.118,* M*_Non-athlete_ = 1.969, *SE* = 0.120, *p* = .553) and 2D-MOT task (*M*_soccer player_ = 2.034, *SE* = 0.115,* M*_Non-athlete_ = 1.798, *SE* = 0.117, *p* = .158). These results also indicate speed ratio threshold in S-3D-MOT of the soccer players group was higher than F-3D-MOT (*p* < .001) and 2D-MOT (*p* < .001), but F-3D-MOT and 2D-MOT were not significantly different from each other (*p* > 0.999). For non-athletes, speed ratio threshold in S-3D-MOT, F-3D-MOT and 2D-MOT did not significantly differ from each other (all *p*s > 0.168). These results indicate the role of binocular 3D cues for MOT speed ratio threshold is essential for soccer players, but not for non-athletes. Further, binocular 3D cues contributed more to the MOT speed ratio thresholds than monocular 3D cues or no 3D cues for soccer players.

There was no significant difference among all the MOT tasks in non-athlete group (*M*_S-3D_ = 2.029, *SE* = 0.138, *M*_F-3D_ = 1.969, *SE* = 0.120,* M*_2D_ = 1.798, *SE* = 0.117, all *p*s > .05). There was also a significant main effect of Modes (*F* (2, 110) = 13.466, *p* < .001, η_p_^2^ = 0.197) and a significant main effect of Groups (*F* (1, 55) = 4.946, *p* = .030, η_p_^2^ = 0.083).

**Fig. 4:**
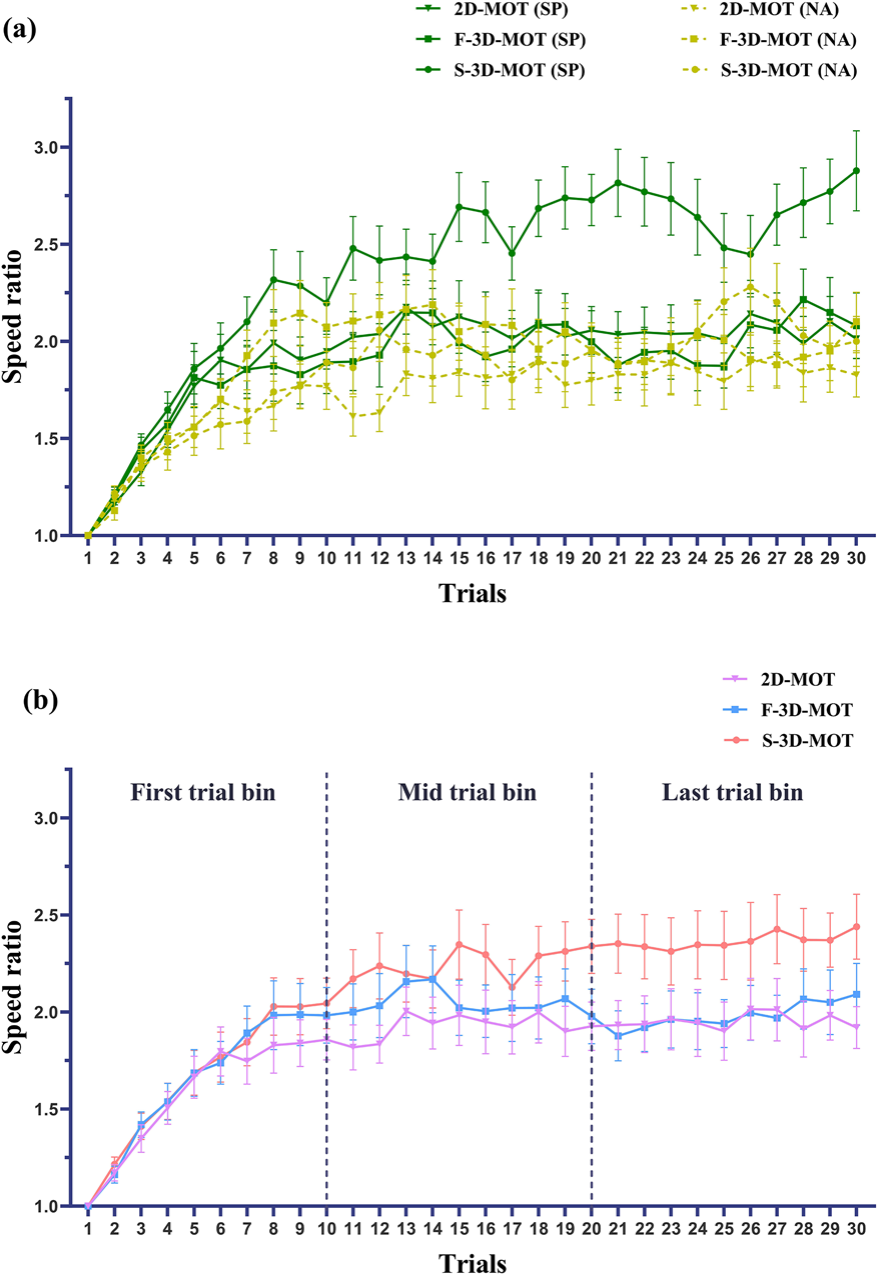
Average tracking speed ratio of three trial bins based on significant two-way interactions: **a** Groups and Modes, and **b** Modes and Trial bins. The abbreviation NA indicates the non-athlete group, and SP indicates the soccer player group. The error bar indicates the SEM of the trial

For the average tracking speed ratio of three trial bins, we submitted 2 Groups (non-athlete, soccer player) × 3 Modes (2D, F-3D, S-3D) × 3 Trial bins (First, Middle, Last) within-between-participants factor repeated-measures ANOVA on average tracking speed. There was a significant interaction of Groups and Modes (*F* (2, 110) = 13.185, *p* < .001, η_p_^2^ = 0.193) on average tracking speed (Fig. 4a). Post-hoc analysis indicated average tracking speed of S-3D-MOT in soccer players’ group (*M* = 2.355, *SE* = 0.091) was significantly higher than non-athlete group (*M* = 1.826, *SE* = 0.092, *p* < .001), but there remain no significantly difference in F-3D-MOT (*M*_Soccer player_ = 1.884, *SE* = 0.092,* M*_NA_ = 1.895, *SE* = 0.094, *p* = .933) and 2D-MOT (*M*_Soccer player_ = 1.921, *SE* = 0.080,* M*_NA_ = 1.717, *SE* = 0.082, *p* = .080). These results indicate findings in line with the speed ratio thresholds above. There was also a significant interaction of Modes and Trial bins (*F* (4, 220) = 4.713, *p* = .001, η_p_^2^ = 0.079) on average tracking speed (Fig. 4b). The post-hoc analysis indicate the average tracking speed of S-3D-MOT (*M* = 2.366, *SE* = 0.093) was significantly higher than 2D-MOT in middle (*M* = 1.928, *SE* = 0.093; *p* = .003) and last period (*M* = 1.952, *SE* = 0.079; *p* < .001), as well as than F-3D-MOT in last period (*M* = 2.047, *SE* = 0.088; *p* < .001). These results indicate the increasing level of average tracking speed is in descending order of both binocular and monocular 3D cues (S-3D-MOT), only monocular 3D cues (F-3D-MOT), and no 3D cues (2D-MOT). The interaction of Groups, Modes, and Trial bins was not significant (*F* (4, 220) = 1.300, *p* = .271, η_p_^2^ = 0.023).

There was also a significant main effect of Trial bins (*F* (2, 110) = 75.960, *p* < .001, η_p_^2^ = 0.580), Modes (*F* (2, 110) = 14.157, *p* < .001, η_p_^2^ = 0.205), and Groups (*F* (1, 55) = 4.865, *p* = .032, η_p_^2^ = 0.081).

## Discussion

This study extends previous research on female youth athletes (Che et al., 2023) by focusing on adult soccer players and non-athletes, thus covering a wider range of cohorts. For soccer players, MOT performance improved with binocular 3D cues (S-3D-MOT) but not with monocular 3D cues (F-3D-MOT). Conversely, non-athletes did not show performance improvements with either type of cue. Moreover, when comparing the two groups, soccer players outperformed non-athletes only in the S-3D-MOT condition, with no differences in other tasks.

### The role of 3D Cues in MOT tracking performance

The current study revealed that S-3D-MOT outperformed F-3D-MOT, while F-3D-MOT did not differ from 2D-MOT in tracking performance. These results are in line with the findings of Zhang et al. (2025). They focused on young athletes (rugby or soccer) with different training loads and demonstrated the beneficial effect of binocular 3D cues on speed thresholds of MOT. The superiority of S-3D-MOT was only evident among soccer players. S-3D-MOT incorporates both additional binocular and monocular 3D cues, unlike classic 2D-MOT. Che et al. (2023) found that S-3D-MOT (referred to as “3D-MOT” in their study) allowed female soccer players to achieve a higher tracking speed threshold. Our results, to some extent, confirm findings from soccer referee offside decision-making tasks. Referees perform better in stereo 3D stimuli conditions than in 2D (Put et al., 2014), as they need to track players’ high-speed movements and defensive players’ positions using MOT-related cognitive processing (Catteeuw et al., 2010). In summary, binocular cues contribute more to MOT tracking performance than monocular cues, highlighting the unique effectiveness of S-3D-MOT for soccer players.

In the non-athlete group, no tracking performance differences were observed with any 3D cues. Regarding monocular 3D cues, our results align with previous non-athlete studies (Hu et al., 2022; Liu et al., 2005; Vidakovic & Zdravkovic, 2010). Liu et al. (2005) found that additional 3D depth frame structures minimally contribute to MOT accuracy. Vidakovic and Zdravkovic (2010) showed that only contrast cues were effective for MOT performance in monocular 3D scenes, not all 3D cues. Hu et al. (2022) also found that monocular 3D self-rotation stimuli in MOT tasks did not outperform 2D stimuli. These studies suggest non-athletes are insensitive to monocular 3D cues. Regarding binocular 3D cues, our findings differ from those of previous studies (Faubert & Allard, 2013; Plourde et al., 2017). Specifically, Plourde et al. (2017) and Faubert and Allard (2013) reported the contribution of binocular 3D cues to multiple object tracking (MOT) performance. The increased task complexity in our study (involving 10 objects and 4 targets, as opposed to 8 objects and 3 targets in Plourde et al., 2017) may have further constrained the attentional resources of non-athletes, thereby amplifying their insensitivity to monocular cues. When tracking speed gradually reaches the threshold under the staircase procedure’s control, tracking multiple high-speed targets is constrained by finite cognitive resources, as limited attention hinders effective allocation to each object. Additionally, more objects and high object motion speed may cause interference between targets and distractors (crowding) (Vater, 2019; Vater et al., 2017), forcing participants to use more attentional resources for selective attention. Most soccer players engage in open-skill sports (Ali et al., 2007) and are thus better at selective attention than non-athletes (Gökçe et al., 2021).

Additionally, average speed ratios across trial bins reveal the temporal trajectory of skill acquisition, showing that soccer players leverage 3D cues to optimize tracking speed earlier in the task, whereas non-athletes struggle with resource allocation in high-load conditions. Soccer players demonstrated faster speed optimization across trials, as evidenced by higher S-3D-MOT performance in the middle and last periods, reflecting their expertise in integrating binocular 3D cues (Welchman et al., 2005). Athletes have stronger functional connectivity between sensory, motor, and cognitive brain regions, supporting more efficient information processing (Wang et al., 2025). Non-athletes may lead to poorer cognitive tasks performance in different 3D depth plane and increased cognitive load (Che et al., 2025). They show less robust connectivity, which may contribute to lower learning efficiency, which may underlie their difficulty in allocating resources during high-load tasks (Gao et al., 2019; Zhang et al., 2024).

This study classified 3D depth cues to investigate their roles. For binocular 3D cues, we were the first to compare their impact on non-athletes’ MOT performance. Non-athletes may not frequently or essentially rely on binocular-cue-based MOT tracking experiences in a stereo environment as much as soccer players. Our results for non-athletes may require further exploration using other MOT indicators to reveal the mechanism of binocular cues in MOT. Thus, future research could examine tracking accuracy indicators for non-athlete groups in the context of the staircase-procedure-derived MOT ability.

### S-3D-MOT and soccer sport

We found that soccer players had an advantage in the S-3D-MOT task, but not in F-3D-MOT and 2D-MOT tasks. This addresses the limitations of previous MOT tasks for soccer players. Meyerhoff et al. (2017) suggested that expertise-related tracking advantages are often limited to closely-matched object-tracking tasks (flat MOT tasks), such as radar control (Allen et al., 2004) and video game (He et al., 2022), rather than team sports (Memmert et al., 2009). However, some studies have shown tracking advantages in team-sport players. Zhang et al. (2009) found that volleyball players outperformed non-athletes in MOT. Qiu et al. (2018) further demonstrated that this advantage is more pronounced in elite-level basketball athletes compared to intermediate-level athletes and non-athletes.

Previous MOT tasks on PC screens lacked the depth perception similar to real-game situations provided by binocular vision (Memmert et al., 2009; Zwierko et al., 2022). Mann et al. (2007) proposed that the measurement sensitivity of perceptual-cognitive skills is positively related to paradigm authenticity in sports. This is further supported by Klostermann and Moeinirad (2020), whose review of 101 studies revealed that expert gaze disparities are most pronounced in tasks with high ecological validity, such as those involving real-world motion and action-perception coupling. Styrkowiec et al. (2024) compared handball players and non-athletes on MOT/MIT tasks and found that athletes employed more target-oriented gaze strategies, despite no group differences in performance accuracy. Together, these findings highlight that while athletes demonstrate specialized gaze behaviors in ecologically realistic settings, performance distinctions hinge on task alignment with sport-specific perceptual demands, underscoring the critical role of stimulus realism in expertise research.

Compared to other MOT tasks, the S-3D-MOT in this study probably provided additional depth perception and more realistic 3D stimuli (Faubert & Sidebottom, 2012; Parsons et al., 2014). Soccer players must rapidly distinguish teammates from opponents in three-dimensional space. The inclusion of binocular depth cues in S-3D-MOT likely enhances this resemblance, as real-game scenarios require constant integration of stereoscopic information (e.g., judging the depth of a teammate’s run or an opponent’s interception angle). The additional binocular depth in S-3D-MOT may be consistent with in-field information processing for soccer players, leading to better performance in S-3D-MOT tasks.

For soccer players, S-3D-MOT ability is a critical cognitive marker with implications for talent identification, as it reflects both innate visual-spatial aptitudes and training-induced adaptations (Li et al., 2024; Yao et al., 2024). While the current study does not differentiate between innate talent and training effects, the task’s ability to distinguish athletes from non-athletes suggests its utility in screening for perceptual-cognitive traits relevant to soccer performance. For instance, talent identification protocols could incorporate S-3D-MOT to assess an athlete’s capacity to integrate binocular depth cues, a skill essential for dynamic decision-making in crowded game scenarios. However, direct evidence linking S-3D-MOT performance to in-field metrics (e.g., pass accuracy, defensive positioning) remains limited (Harenberg et al., 2021; Romeas et al., 2025; Vater et al., 2021). Future longitudinal studies should test whether S-3D-MOT training enhances on-field performance metrics. In contrast, non-athletes lack both the cognitive foundation and sport-specific demands to utilize 3D cues effectively, highlighting the task’s specificity to athletic populations.

### Potential visual processing mechanisms in soccer players

The better S-3D-MOT performance of soccer players compared to non-athletes may be attributed to differences in stereovision. Open-skill sports like football and basketball have high demands for stereovision due to frequent unpredictable environmental changes (Boden et al., 2009; Presta et al., 2021). Buys and Ferreira (2008) reported that over half of the athletes (52.5%) achieved the highest stereovision scores, indicating top athletes’ excellent stereovision skills. However, there is debate about whether stereovision can be improved through training, which is consistent with Vater et al.’s (2021) critical view on the transfer of S-3D-MOT training to soccer performance. Romeas et al. (2025) found that while dual-task 3D-MOT training improved task-specific speed thresholds in youth soccer athletes, these improvements did not transfer to other tasks. Thus, S-3D-MOT-related stereovision may serve as an evaluation tool for team-sport players due to their long-term practice, but the training effect of S-3D-MOT for soccer players may be overestimated. One possible mechanism is that field practice/performance and S-3D-MOT tracking thresholds may not interact reciprocally, with the relationship only going from field practice/performance to S-3D-MOT tracking performance.

Our study not only replicates previous findings of Che et al. (2023) but also extends them by demonstrating that soccer players, including both male and female adult athletes, better utilize binocular 3D cues in S-3D-MOT compared to monocular 3D cues or non-3D cues in other MOT tasks, with this advantage being significantly more pronounced than in non-athletes.

### Limitations and future directions

This study found that soccer players had a higher speed-ratio threshold than non-athletes. While causal links between S-3D-MOT and real-world performance require further validation, the task’s reliance on stereovision and selective attention—both critical for soccer—suggests it captures essential perceptual-cognitive mechanisms. This positions S-3D-MOT as a valuable tool for talent identification and baseline skill assessment. While S-3D-MOT shows promise as a diagnostic tool for talent identification, its utility as a training intervention requires cautious interpretation. Current evidence suggests task-specific improvements (e.g., speed thresholds) but limited transfer to broader performance domains (Harris et al., 2020; Romeas et al., 2025). Coaches should integrate S-3D-MOT with complementary assessments (e.g., tactical decision-making in VR environments) to holistically evaluate athletes’ perceptual-cognitive readiness (Romeas et al., 2019).

One limitation is that the 2D-MOT condition lacked cross-overs, and the higher attentional load (10 objects, 4 targets) in this study compare to Plourde et al. (2017) (8 objects, 3 targets) might have influenced results, thereby potentially limiting the conclusion that monocular depth cues are irrelevant to the task. The relationship between tracking objects and motion speed is complex, as video-gaming experts can track more targets (Green & Bavelier, 2006). Thus, the impact of varying tracking target numbers in S-3D-MOT for soccer players remains unclear. Future research should systematically manipulate the number of targets (e.g., 3–6 targets) to investigate how attentional load influences S-3D-MOT performance in soccer players. Object size affects MOT tracking performance, with small objects hindering performance (Fehd & Seiffert, 2010). Given soccer’s large field and small visual angles in long-pass scenarios, further research is needed to understand its effect on S-3D-MOT performance.

A limitation of this study is not measure stereoacuity. Previous research indicates that athletes may have better stereoscopic vision (Boden et al., 2009; Laby et al., 1996), which could explain their superior S-3D-MOT performance. Future research should address this. Additionally, the observed S-3D-MOT advantage in soccer players may reflect both training-induced adaptations and innate abilities. To clarify this, future studies should include stratified samples (e.g., novices, intermediates, elites) and control groups from other sports, enabling systematic comparisons of skill development trajectories*.*

## Conclusion

Soccer players exhibited better tracking performance in S-3D-MOT than non-athletes. Soccer players are more sensitive to binocular cues provided by S-3D-MOT than monocular cues provided by F-3D-MOT or non 3D cues provided by classic 2D-MOT. This study highlighted the advantages and applicability of S-3D-MOT for talent identification and evaluation of team sports players, such as soccer.

## Data Availability

The datasets used and/or analysed during the current study are available from the corresponding author upon reasonable request.
